# GnRHa Triggering Versus hCG Triggering in PCOS Patients Who Undergo Fresh or FET Cycles: Is the King Fake or Real?

**DOI:** 10.3390/medicina61122195

**Published:** 2025-12-11

**Authors:** Muserref Banu Yilmaz, Reyyan Gokcen Iscan, Sevdenur Banu Yigit, Esra Tustas Haberal, Belgin Devranoglu, Ayse Nur Aksoy, Ali Irfan Guzel, Pinar Kumru

**Affiliations:** 1 Department of Obstetrics and Gynecology, Zeynep Kamil Women and Children Diseases Training and Research Hospital, University of Health Sciences, 34668 Istanbul, Türkiye; reyyangokcen@gmail.com (R.G.I.); sevdenuryigit55@gmail.com (S.B.Y.); bdevranoglu@superonline.com (B.D.); pkumru@gmail.com (P.K.); 2 Hisar Hospital, 34768 Istanbul, Türkiye; etustas@hotmail.com; 3 Department of Obstetrics and Gynecology, Erzurum City Hospital, University of Health Sciences, 25070 Erzurum, Türkiye; draysenuraksoy@hotmail.com; 4 School of Medicine, Sanko University, 27090 Gaziantep, Türkiye; aliirfanguzel77@gmail.com

**Keywords:** GnRHa triggering, hCG triggering, PCOS, IVF, infertility, cycle outcome

## Abstract

*Background and Objectives*: To evaluate the impact of gonadotropin-releasing hormone agonist (GnRHa) triggering compared to human chorionic gonadotropin (hCG) triggering on frozen–thawed and fresh embryo transfer outcomes in patients with polycystic ovary syndrome (PCOS) undergoing in vitro fertilization (IVF). *Materials and Methods*: This retrospective cohort study analyzed 267 IVF cycles of 261 PCOS patients treated with GnRH antagonist protocols. Patients were divided into three groups: GnRHa-triggered frozen–thawed embryo transfer (ET) (*n* = 126), hCG-triggered frozen–thawed ET (*n* = 68), and hCG-triggered fresh ET (*n* = 73). Baseline characteristics, stimulation parameters, and cycle outcomes were compared between groups. A binary logistic regression analysis was established to identify independent predictors of clinical pregnancy. *Results*: The GnRHa-triggered group had significantly higher numbers of retrieved oocytes, mature (MII) oocytes, and fertilized oocytes compared to both hCG-triggered groups (*p* < 0.001). The number of obtained embryos and frozen embryos (good-quality embryos) was highest in the GnRHa group (*p* < 0.001). However, clinical pregnancy rates were comparable between the groups with a similar number and grade of transferred embryos (32.53%, 38.23%, and 32.87%, respectively). Multivariate regression analysis revealed that the grade of the transferred embryo was a significant predictor of clinical pregnancy (*p* = 0.034). *Conclusions*: This study provides insights into different triggering strategies for final oocyte maturation in PCOS patients. GnRH-agonist-triggered frozen–thawed cycles showed comparable clinical pregnancy outcomes to those of hCG-triggered cycles, with a potentially lower OHSS risk. The findings suggest that individualized triggering approaches based on patient characteristics and OHSS risk may be beneficial for PCOS patients undergoing IVF.

## 1. Introduction

Polycystic ovary syndrome (PCOS) is the most common endocrine disorder in women of reproductive age, with a prevalence ranging from 5% to 13% [1]. It is the leading cause of anovulatory infertility and is characterized by hyperandrogenism, oligo-anovulation, and polycystic ovarian morphology, often accompanied by clinical and metabolic comorbidities [2]. In vitro fertilization (IVF) is frequently required as the final treatment step for infertile PCOS patients. However, these patients are at a particularly high risk of ovarian hyperstimulation syndrome (OHSS) due to their typically high follicle count [3,4]. To reduce this risk, lower gonadotropin doses and pituitary suppression with gonadotropin-releasing hormone (GnRH) antagonists are commonly used during ovarian stimulation [5].

The choice of trigger agent for final oocyte maturation also plays a critical role in reducing the risk of OHSS. Human chorionic gonadotropin (hCG), traditionally used for this purpose, mimics endogenous luteinizing hormone (LH). However, because hCG has a longer half-life than LH, it exerts a prolonged luteotrophic effect that significantly increases the risk of OHSS [6]. Moreover, supraphysiological luteal estradiol and progesterone levels associated with this LH-like effect have been reported to impair both oocyte quality and endometrial receptivity [7,8].

To address these disadvantages, the use of a gonadotropin-releasing hormone agonist (GnRHa) trigger has been proposed as a physiological alternative. By binding to pituitary GnRH receptors, GnRHa induces a surge in gonadotropins (flare-up effect), leading to final oocyte maturation [9]. Early studies showed that GnRHa triggers are highly effective in preventing OHSS, particularly in high-risk populations such as women with PCOS. However, subsequent studies reported lower live birth rates in fresh transfer cycles following GnRHa triggering compared to hCG, primarily due to luteal phase insufficiency that could not be fully corrected with standard luteal support [10,11,12]. Consequently, the freeze-all strategy has become the preferred approach for GnRHa-triggered cycles, allowing for embryo transfer in subsequent frozen–thawed cycles [13,14].

Given this context, the present study aimed to evaluate the impact of GnRHa triggering on frozen–thawed embryo transfer outcomes in patients with PCOS, and to compare these results with those of cycles triggered by hCG.

## 2. Materials and Methods

### 2.1. Study Design and Participants

This retrospective cohort study was conducted on infertile women with PCOS treated with a GnRH antagonist Controlled Ovarian Stimulation (COH) protocol for IVF, at Assisted Reproductive Unit of Zeynep Kamil Women and Children Diseases Training and Research Hospital of University of Health Sciences, Istanbul, Türkiye. Medical records from January 2019 to December 2024 were reviewed.

Eligible participants were women aged 18–40 years diagnosed with PCOS according to the Rotterdam Consensus criteria, requiring at least two of the following three features: (1) oligoovulation or anovulation, (2) ultrasonographic evidence of polycystic ovaries, and (3) clinical or biochemical hyperandrogenism, after exclusion of other endocrine disorders, such as congenital adrenal hyperplasia and hypothyroidism [2].

Patients under the age of 18 and over the age of 40, co-existing male factors, and with a history of systemic disease (diabetes, insulin resistance, hypertension, etc.) or chronic medication use were excluded. In addition, patients whose transfer was canceled due to poor embryo development were eliminated from the study.

### 2.2. Data Collection and Grouping

Baseline characteristics of age, duration of infertility, basal hormonal assessments, antral follicle counts, anti-Mullerian hormone (AMH) levels, cycle characteristics, treatment modalities, number and quality of embryos transferred, and pregnancy outcomes were collected from the files. Patients were classified based on the type of trigger used for oocyte maturation and the type of embryo transfer as follows:

Group A: Patients triggered by GnRHa + frozen–thawed embryo transfer

Group B: Patients triggered by hCG + frozen–thawed embryo transfer

Group C: Patients triggered by hCG + fresh embryo transfer

In patients triggered with a GnRH agonist, fresh embryo transfer was not performed due to the risk of luteal phase insufficiency, which is a well-recognized limitation of GnRHa triggering in the absence of intensive luteal support [15].

### 2.3. IVF Protocol

Before stimulation, all patients were evaluated with ultrasonography, and basal FSH, LH, E2, and anti-mullerian hormone (AMH) levels were measured during the early follicular phase.

All patients underwent COH using a multidose GnRH antagonist protocol after confirmation of the absence of infection by cervicovaginal culture. On the second or third day of menstruation, recombinant FSH (Gonal-F^®^, Serono, Geneva, Switzerland), ranging from 112.5 to 150 IU daily, was started with an individualized dose taking into consideration the patient’s body mass index (BMI), age, ovarian reserve, and previous ovarian responses to stimulation. Four days after starting gonadotropin, ovarian response was evaluated using transvaginal ultrasonography and serum E2 levels. The adjusted gonadotropin dose was continued, and a 0.25 mg dose of GnRH antagonist (Cetrotide^®^, Serono, Switzerland) was added subcutaneously from the day that the diameter of the leading follicle reached ≥12 mm until the day of the trigger. When leading follicles reached ≥17 mm in diameter, oocyte maturation was triggered. Patients who were considered at high risk of developing severe OHSS (E2 ≥ 3000 pg/mL and/or ≥15 follicles ≥17 mm during ovarian stimulation) were triggered with 0.2 mg triptoreline acetate (Gonapeptyl^®^; Ferring, Saint-Prex, Switzerland) for final oocyte maturation (GnRH agonist group). Coasting was not performed in any patient. Other patients who were considered at low risk of developing OHSS were triggered with 250 µg choriogonadotropin alpha (Ovitrelle^®^; Serono, Switzerland).

Transvaginal ultrasound-guided oocyte pick-up was performed 35 h after triggering under intravenous sedation using a single-lumen needle. After the routine expectation period of each oocyte and denudation, intracytoplasmic sperm injection (ICSI) was performed in all cases. Fertilization assessments were performed 24 h after ICSI, and embryos were evaluated according to the Gardner–Schoolcraft embryo grading system [16]. Embryos were evaluated on their cleavage stage, for having an appropriate cell number for their developmental stage, uniform and regularly shaped blastomeres, and minimal cytoplasmic fragmentation. High-quality embryos (Grade 1) were defined as those demonstrating symmetrical blastomeres, ≤10% fragmentation, and absence of multinucleation. For blastocysts, this system assesses embryos according to three main components: the degree of blastocyst expansion, the quality of the inner cell mass (ICM), and the quality of the trophectoderm (TE), the latter two scored from A to C. Grade 1 embryos were defined as blastocysts with an expansion grade of ≥3 and with both ICM and TE graded as A or B.

Patients triggered by GnRHa underwent total embryo freezing (Group A). All-freeze was also planned for patients triggered by hCG whose endometrial receptivity was considered inadequate (endometrial thickness < 7 mm or a heterogeneous endometrial sonographic pattern on the day of hCG triggering) (Group B). Patients triggered by hCG who had adequate endometrial receptivity underwent embryo transfer in the same cycle (Group C).

### 2.4. Embryo Transfer and Luteal Support

All patients were evaluated for early OHSS, according to American Society for Reproductive Medicine (ASRM) guidelines, between days 2 and 6 following oocyte collection with complete blood count (CBC), physical examination, and ultrasound assessment [17]. Patients with high risk of OHSS were prescribed 0.5 mg daily cabergoline (Dostinex^®^, Pfizer, Roma, Italy) starting on the day of oocyte pick-up (OPU) and continuing for one week. In fresh embryo transfer cycles, luteal phase supplementation was provided by daily administration of 600 mg vaginal micronized progesterone (Progestan^®^; Kocak Farma, Istanbul, Turkey) from the day of OPU until either a negative pregnancy test or 12 weeks of gestation. Embryos that were evaluated as good-quality among those remaining after fresh embryo transfer were cryopreserved.

In frozen embryo transfer cycles, endometrial preparation was performed with hormone replacement therapy after confirming downregulation in the early follicular phase. A standard oral dose of 6 mg estrogen (Estrofem^®^; Novo Nordisc, Bagsvaerd, Denmark) was administered until day 12 of the cycle. When endometrial thickness reached 8–14 mm, 600 mg vaginal with 25 mg subcutaneously micronized progesterone (Progestan^®^ and Progestan Dex^®^; Kocak Farma, Turkey) was added. Progesterone administration was continued according to the age of the embryo.

The number of embryos transferred was chosen according to embryo quality and the patients’ previous cycle characteristics. Cleavage-stage embryos were transferred after 2–4 days of progesterone administration, and blastocysts were transferred after 5 days of progesterone administration [18]. All embryo transfers were performed under abdominal ultrasound guidance 2–3 h after thawing the embryos.

### 2.5. Pregnancy Assessment

The pregnancy status of the patients was determined by measuring β-hCG on the 12th day of transfer for the cleavage stage and the 10th day of transfer for the blastocyst stage. The situation where the β-hCG value is above 5 IU/L was considered a ‘positive pregnancy’, and was accepted as a ‘biochemical pregnancy’ when the value exceeded 40 IU/L. After monitoring hCG doubling and visualization of the intrauterine gestational sac by ultrasonography, confirming fetal heartbeat 5–6 weeks after transfer is accepted as a ‘clinical pregnancy.’ Pregnancy rates were calculated after the first frozen–thawed transfer of frozen groups.

### 2.6. Ethical Permission

The study protocol was approved by the Ethical Committee of Health Sciences University Zeynep Kamil Women and Children Health Training and Research Hospital on 24 November 2021 (approval number 186/2021) and revised to extend the duration to 28 May 2025. The study was conducted in accordance with the principles of the Declaration of Helsinki (2013 revision) and relevant national and institutional regulations. Written informed consent was not obtained from the patients, as the retrospective design of the study rendered it unnecessary in accordance with the regulations of the ethics committee.

### 2.7. Statistical Analysis

SPSS 20 software (SPSS Inc., Chicago, IL, USA) was used for statistical analysis of data. The normality of the variables was analyzed using the Kolmogorov–Smirnov test. The mean values of the three groups were analyzed using one-way ANOVA followed by the post hoc Bonferroni test for multiple comparisons. The sample size was determined according to the results of the Central Limit Theorem, which indicated that we needed at least 30 individuals in each subgroup [19]. Two-sided *p*-values were considered statistically significant at *p* < 0.05. Data are presented as median values ± standard deviation (SD) or number and percentage values.

A subgroup analysis was performed to compare the baseline characteristics, stimulation parameters, and cycle outcomes between patients who achieved clinical pregnancy and those who did not. Continuous variables were analyzed using the independent samples *t*-test (or the Mann–Whitney U test if the data were not normally distributed), while categorical variables were evaluated using the chi-square test. Furthermore, a binary logistic regression analysis was established to identify independent predictors of clinical pregnancy. Among the variables tested, maternal age and transferred embryo grade were included in the model with the type of trigger and the mode of embryo transfer, which were the main features defining the study groups.

## 3. Results

**Figure 1:**
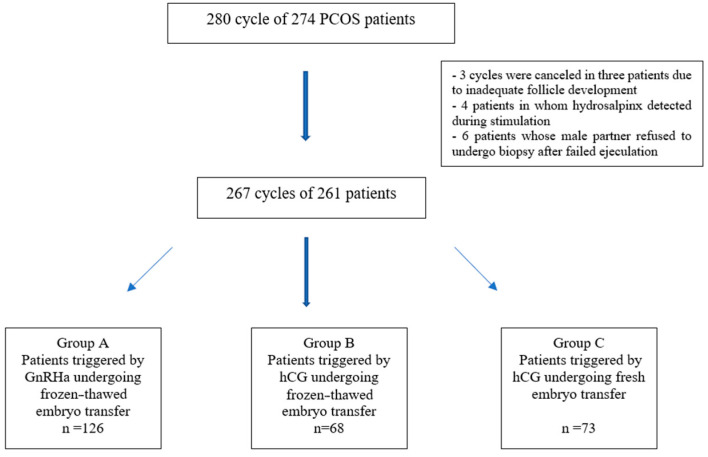
Flowchart of the study population enrollment.

Two hundred and eighty cycles of 274 PCOS patients stimulated for IVF were enrolled in the study. Cycles were canceled in three patients due to inadequate follicle development, four patients in whom hydrosalpinx was detected during stimulation, and six patients whose male partner refused to undergo biopsy after failed ejaculation were excluded from the study. Thus, 267 cycles of 261 patients were included in the main analysis. In 128 cycles, patients were triggered by GnRHa, and in 68 cycles, patients were triggered by hCG and underwent frozen–thawed embryo transfer. In 73 cycles in which patients were triggered by hCG, a fresh embryo transfer was performed. The flowchart is shown in Figure 1.

When the baseline characteristics among the groups were compared, the mean age was significantly lower in the frozen ET-GnRH agonist group than in both the frozen ET-hCG and fresh ET-hCG groups (*p* < 0.001). The duration of infertility and basal FSH levels were comparable across groups (*p* > 0.05). However, significant differences were observed in LH, E2, and AMH levels, with the highest values detected in the frozen ET agonist group (*p* < 0.001). The antral follicle count (AFC) was similar among the three groups (*p* > 0.05) (Table 1).

The stimulation parameters and cycle outcomes of the participants are summarized in Table 2. Stimulation time, total gonadotropin dose, and antagonist duration did not differ significantly among the groups (*p* > 0.05). Estradiol levels on the trigger day were significantly higher in the frozen ET-GnRH agonist group compared with both the frozen ET-hCG and fresh ET-hCG groups (*p* < 0.001). Similarly, the total number of retrieved oocytes, MII oocyte number, MII oocyte ratio, and the number of fertilized oocytes were all significantly higher in the frozen ET-GnRH agonist group (*p* < 0.001).

The number of obtained embryos was significantly greater in the frozen ET-GnRH agonist group compared to the frozen ET-hCG and fresh ET-hCG groups (*p* < 0.001). Likewise, the number of frozen embryos was significantly higher in the frozen ET-GnRH agonist group, followed by the frozen ET-hCG group, with the lowest numbers observed in the fresh ET-hCG group (*p* < 0.001). Consistent with the comparable fertilization rates, the number and the grade of embryos transferred did not show significant differences between the groups (*p* > 0.05). Also, clinical pregnancy rates were similar between the groups (32.53%, 38.23%, and 32.87%, respectively).

No cases of moderate or severe OHSS were observed in either group; only two patients who conceived in the fresh ET-hCG group developed late-onset OHSS, which was managed conservatively.

In the subgroup analysis, which was performed to compare the patients who achieved and did not achieve clinical pregnancy, no significant differences were observed in terms of baseline characteristics, stimulation parameters, or cycle outcomes. The only parameter showing a statistically significant difference was the grade of the transferred embryos, which was higher in patients who achieved clinical pregnancy (*p* = 0.035). These results are presented in Supplementary Table S1.

Multivariate regression analysis, including the main variables characterizing the groups, embryo grade, and patient age, revealed that the grade of transferred embryo was a significant predictor of clinical pregnancy (*p* = 0.034), indicating that higher-quality embryos were associated with increased pregnancy rates. In contrast, age, trigger type, and embryo transfer method did not show a statistically significant association with clinical pregnancy outcomes (*p* > 0.05) (Table 3).

## 4. Discussion

Human chorionic gonadotropin (hCG) has historically been the standard agent for final oocyte maturation in IVF cycles; however, its prolonged luteotropic effect substantially increases the risk of ovarian hyperstimulation syndrome (OHSS), especially in women with PCOS, who often present with high follicular counts. In contrast, gonadotropin-releasing hormone agonist (GnRHa) triggering induces a short-lived but physiological luteinizing hormone surge, making it a safer option for hyperresponders [5,6,7,8,9].

The present study compared IVF outcomes in PCOS patients undergoing different triggering and embryo transfer strategies. Our results demonstrated that the GnRHa trigger was associated with a significantly higher yield of mature (MII) oocytes and fertilized oocytes compared with hCG triggering. Also, we found higher maturation and fertilization rates with GnRHa than hCG, which were independent outcomes from group characteristics.

The number of obtained embryos was significantly greater in the GnRHa-triggered group compared to the hCG-triggered frozen and fresh groups. Similarly, the number of frozen embryos—which meant Grade 1 since our ART clinic, as a principle, only freezes good-quality embryos—was highest in the GnRHa group, followed by the hCG-frozen group, with the lowest numbers in the hCG-fresh group. Although comparing the fresh embryo transfer group, in which only the remaining good-quality embryos were cryopreserved, may not be entirely appropriate, this result indicates that a higher number of top-quality (Grade 1) embryos were obtained in the agonist group compared to the hCG group.

The subgroup analysis, which was performed to compare all parameters for clinical pregnancy, revealed no significant differences in cycle characteristics and outcome other than embryo grade. According to the logistic regression analysis, the grade of transferred embryo was a significant predictor of clinical pregnancy, meaning each one-unit increase in embryo grade was associated with an approximately 46% decrease in the likelihood of achieving clinical pregnancy. Consistent with the literature, this result indicates that embryo quality emerged as the key determinant of success.

Recent evidence suggests that while GnRHa triggering effectively prevents OHSS, outcomes may vary depending on luteal support and transfer strategy. Wang et al. reported comparable fertilization rates and pregnancy outcomes between GnRHa-only and dual-trigger approaches in PCOS patients undergoing a freeze-all strategy, although GnRHa resulted in a higher number of retrieved oocytes [20]. Similarly, Krishna et al. and Elarab et al. found that GnRHa reduced the incidence of OHSS without compromising embryo quality or pregnancy rates in antagonist cycles [21,22]. A systematic review emphasized that optimal luteal support or freeze-all strategies are essential to maintain reproductive outcomes when GnRHa is used [23].

According to our data, although it was not significant, clinical pregnancy rates were higher in the hCG-triggered frozen–thawed group compared to both the GnRHa-triggered frozen group and the hCG-triggered fresh group, despite similar transferred number and grade of embryos. This result may reflect subtle differences in endometrial receptivity or embryo–endometrium synchrony, which remains an area of ongoing investigation. Recent studies have questioned whether maximizing oocyte yield is always beneficial, suggesting that embryo competence, rather than quantity, should guide clinical decision-making [24,25]. These findings align with our results, affirming that GnRHa triggering improves oocyte and embryo output and enhances cumulative reproductive success when FET is utilized.

The discrepancy between embryo yield and clinical pregnancy outcomes suggests that maximizing oocyte numbers may not always translate into improved pregnancy rates. Endometrial factors, timing of transfer, and luteal support protocols are critical considerations. Our data emphasize the need to balance embryo quantity with transfer strategy and endometrial receptivity, tailoring approaches for PCOS patients to maximize success while minimizing risk.

### Strengths and Limitations

The strengths of our study include the evaluation of a well-defined PCOS population and the comparison of both fresh and frozen–thawed transfers across different triggers. A limitation of this study is its retrospective and non-randomized design, as patients were allocated to triggering and transfer strategies according to their baseline risk of OHSS rather than by random assignment. This allocation approach may have introduced selection bias and could partly account for the observed differences in oocyte and embryo outcomes, independent of the triggering method. However, we attempted to reduce this potential bias through subgroup and regression analyses, which suggested methodological comparability between the groups was largely maintained and that the observed trends in pregnancy outcomes were unlikely to be solely driven by patient selection.

The retrospective design also limited us to comparing the groups according to hormonal or metabolic parameters. The relatively small sample size and the lack of follow-up of the pregnancies until delivery are the other limitations of the study. Therefore, studies with larger patient populations and prospective case–controlled designs are needed to obtain more conclusive results. Future randomized controlled trials with long-term follow-up, including live birth and obstetric outcomes, are warranted to clarify the optimal trigger and transfer strategies in patients with PCOS.

## 5. Conclusions

In conclusion, this study provides valuable insights into the efficacy and safety of different triggering strategies for final oocyte maturation in patients with PCOS undergoing IVF. The results demonstrate that GnRH agonist-triggered frozen–thawed cycles yield comparable clinical pregnancy outcomes to hCG-triggered cycles, while potentially offering a safer profile in terms of OHSS risk. In particular, the variability in pregnancy rates among PCOS patients despite a high oocyte yield supports the notion that not only the number of oocytes, but also embryo quality and endometrial receptivity (embryo–endometrium synchronization), play a crucial role in achieving a successful pregnancy.

These findings underscore the importance of individualized treatment approaches for PCOS patients, considering factors such as OHSS risk, endometrial receptivity, and embryo quality. While this study has limitations, including its retrospective nature, it contributes to the growing body of evidence supporting the use of GnRH agonist triggering as a viable alternative to hCG in high-risk PCOS patients. Future prospective studies are warranted to elucidate the optimal triggering and transfer strategies for maximizing reproductive outcomes in this patient population.

## Figures and Tables

**Table 1:** Comparison of baseline characteristics of PCOS patients triggered with GnRHa and hCG.

Variables	Frozen ET-GnRH Agonist ^a^ (*n* = 126) $\bar{x}\pm SD$	Frozen ET-hCG ^b^ (*n* = 68) $\bar{x}\pm SD$	Fresh ET-hCG ^c^ (*n* = 73) $\bar{x}\pm SD$	F	p *	Bonferroni Test (*p*) *
Age (years)	27.7 ± 3.3	31.4 ± 2.6	28.8 ± 4.3	449.96	<0.001	a–b *p* < 0.001 a–c *p* < 0.001 b–c *p* < 0.001
Duration † (months)	76.12 ± 8.44	77.21 ± 6.67	82.14 ± 10.01	66.34	<0.001	a–b *p* > 0.005 a–c *p* > 0.005 b–c *p* > 0.005
FSH (IU/mL)	6.31 ± 2.34	5.42 ± 2.39	5.66 ± 3.54	16.91	>0.001	a–b *p* > 0.005 a–c *p* > 0.005 b–c *p* > 0.005
LH (IU/mL)	8.18 ± 2.88	5.28 ± 1.66	4.90 ± 3.22	22.24	<0.001	a–b *p* < 0.001 a–c *p* < 0.001 b–c *p* < 0.001
E2(pg/mL)	50.36 ± 12.64	54.45 ± 11.78	40.53 ± 9.65	27.39	<0.001	a–b *p* < 0.001 a–c *p* < 0.001 b–c *p* < 0.001
AFC	17.61 ± 3.05	18.33 ± 4.08	17.76 ± 5.33	16.85	>0.001	a–b *p* > 0.005 a–c *p* > 0.005 b–c *p* > 0.005
AMH (pg/mL)	6.32 ± 2.22	4.75 ± 1.65	4.19 ± 1.84	24.55	<0.001	a–b *p* < 0.001 a–c *p* < 0.001 b–c *p* < 0.001

a: **Frozen ET-GnRH agonist trigger;** b: **Frozen ET-hCG trigger;** c: **Fresh ET-hCG trigger;** hCG: human chorionic gonadotropin; Sd: standard deviation; †: duration of infertility; FSH: follicle stimulating hormone; LH: luteinizing hormone; E2: estradiol hormone; AFC: antral follicle count; AMH: anti-mullerian hormone, *: *p* < 0.05.

**Table 2:** Comparison of stimulation parameters and cycle outcomes of the PCOS patients triggered with GnRHa and hCG.

Variables	Frozen ET-Agonist ^a^ (*n* = 126) $\bar{x}\pm SD$	Frozen ET-hCG ^b^ (*n* = 68) $\bar{x}\pm SD$	Fresh ET-hCG ^c^ (*n* = 73) $\bar{x}\pm SD$	F	p *	Bonferroni Test (*p*) *
Stimulation Time (Day)	9.27 ± 3.23	10.02 ± 4.67	9.53 ± 5.87	458.86	>0.001	a–b *p* > 0.005 a–c *p* > 0.005 b–c *p* > 0.005
Total Gonadotropin Dose (IU)	1862.374 ± 433.23	2165.38 ± 566.98	1984.26 ± 488.45	64.22	>0.001	a–b *p* > 0.005 a–c *p* > 0.005 b–c *p* > 0.005
Antagonist Duration	5.18 ± 2.43	4.47 ± 1.77	5.05 ± 2.54	43.91	>0.001	a–b *p* > 0.005 a–c *p* > 0.005 b–c *p* > 0.005
Trigger Day E2 (pg/mL)	2544.12 ± 552.92	1861.82 ± 484.33	1515.49 ± 325.32	32.54	<0.001	a–b *p* < 0.001 a–c *p* < 0.001 b–c *p* < 0.001
Total Oocyte Number	13.11 ± 4.65	10.94 ± 6.12	8.15 ± 5.44	17.33	<0.001	a–b *p* < 0.001 a–c *p* < 0.001 b–c *p* < 0.001
MII Oocyte Number	12.12 ± 3.45	8.11 ± 6.22	7.01 ± 3.70	77.85	<0.001	a–b *p* < 0.001 a–c *p* < 0.001 b–c *p* < 0.001
MII Oocyte Ratio (%)	94	82	91	86.44	<0.001	a–b *p* < 0.001 a–c *p* < 0.001 b–c *p* < 0.001
Fertilized Oocyte Number	8.66 ± 5.24	6.02 ± 4.12	4.22 ± 2.11	56.88	<0.001	a–b *p* < 0.001 a–c *p* < 0.001 b–c *p* < 0.001
Fertilization Ratio (%)	71	61	58	22.51	>0.001	a–b *p* > 0.005 a–c *p* > 0.005 b–c *p* > 0.005
Number of Obtained Embryos	5.40 ± 1.80	4.60 ± 1.22	3.26 ± 1.01	24.54	<0.001	a–b *p* < 0.001 a–c *p* < 0.001 b–c *p* < 0.001
Number of Frozen Embryos	3.40 ± 0.99	3.07 ± 1.32	1.50 ± 0.89	33.78	<0.001	a–b *p* < 0.001 a–c *p* < 0.001 b–c *p* < 0.001
Number of Embryos Transferred	1.04 ± 0.45	1.11 ± 1.02	1.12 ± 0.56	37.93	>0.001	a–b *p* > 0.005 a–c *p* > 0.005 b–c *p* > 0.005
Grade of Embryos Transferred	1.33 ± 1.10	1.29 ± 0.78	1.41 ± 1.06	19.25	>0.001	a–b *p* > 0.005 a–c *p* > 0.005 b–c *p* > 0.005
Clinical Pregnancy Rates (%)	32.53	38.23	32.87	43.22	>0.001	a–b *p* > 0.005 a–c *p* > 0.005 b–c *p* > 0.005

a: **Frozen ET-GnRH agonist Trigger;** b: **Frozen ET-hCG Trigger;** c: **Fresh ET-hCG Trigger;** hCG: human chorionic gonadotropin; E2: estradiol; MII: metaphase 2. Values were presented as mean ± SD or %, *: *p* < 0.05.

**Table 3:** Multivariate regression analysis established for positive pregnancy using the variables of age, embryo grade, type of triggering, and mode of embryo transfer.

Constant Parameters	Unstandardized Coefficients		Standardized Coefficients (β)	*p* Values *
	B	SE		
Age	0.020	0.029	1.020	0.499
Grade of Embryos Transferred	−0.625	0.295	0.535	0.034
Trigger Type (GnRHa vs. hCG)	0.0228	0.317	1.257	0.471
Mode of ET (Frozen vs. Fresh)	−0.193	0.358	0.824	0.589

hCG: human chorionic gonadotropin; ET: embryo transfer; *: *p* < 0.05.

## Data Availability

The data that support the findings of this study are not publicly available due to protect the privacy of participants, but they are available on request from the corresponding author.

## Supplementary Materials

The following supporting information can be downloaded at: https://www.mdpi.com/article/10.3390/medicina61122195/s1, Table S1: Comparison of baseline characteristics, stimulation parameters, and cycle outcomes between patients who achieved and not achieved clinical pregnancy.
