# Supplementary material for: GnRHa Triggering Versus hCG Triggering in PCOS Patients Who Undergo Fresh or FET Cycles: Is the King Fake or Real?
Source: Medicina (Kaunas). 2025 Dec 11;61(12):2195. doi: 10.3390/medicina61122195 (PMC12734809; doi:10.3390/medicina61122195)
Supplement: Supplementary file 1 [file medicina-61-02195-s001.zip › medicina-3980359-supplementary.pdf]

Supplementary Table S1. Comparison of baseline characteristics, stimulation parameters, and cycle outcomes between patients who achieved and not achieved clinical pregnancy.

Variables	**Negative Pregnancy** (n=176) $\bar{x} \pm SD$	**Positive Pregnancy** (n=91) $\bar{x} \pm SD$	**P values***
Age (years)	31.1 ± 4.4	31.2 ± 4.8	0.519
Duration*(months)	63.5 ± 37.1	68.0 ± 33.9	0.724
FSH (IU/mL)	5.7 ± 1.5	5.8 ± 2.0	0.886
LH (IU/mL)	8.2 ± 5.8	7.4 ± 6.7	0.183
E2(pg/mL)	49.4 ± 28.6	47.4 ± 32.3	0.438
AFC	14.4 ± 5.8	15.5 ± 6.0	0.512
AMH (pg/ml)	3.2 ± 5.6	2.9 ± 3.0	0.232
Stimulation Time (day)	9.6 ± 2.0	9.4 ± 1.7	0.528
Total Gonadotropin Dose (IU)	2063.0 ± 902.0	2062.1 ± 908.7	0.992
Antagonist Duration	5.3 ± 1.6	5.2 ± 1.3	0.585
Trigger Day E2 (pg/mL)	2564.0 ± 1834.7	2220.0 ± 1009.2	0.336
Total Oocyte Number	14.5 ± 9.1	15.7 ± 8.8	0.245
MII Oocyte Number	10.4 ± 6.4	11.3 ± 6.3	0.230
MII Oocyte Ratio (%)	73.8 ± 17.9	74.3 ± 16.7	0.907
Fertilized Oocyte Number	7.0 ± 4.5	7.8 ± 4.7	0.152
Fertilization Ratio (%)	70.0 ± 21.0	70.2 ± 22.1	0.776
Number of Obtained Embryos	4.6 ± 3.0	5.0 ± 3.1	0.273
Number of Frozen Embryos	2.7 ± 2.5	3.0 ± 2.9	0.364
Number of Embryos Transferred	1.2 ± 0.4	1.3 ± 0.5	0.471

Grade of Embryos Transferred	1.4 ± 0.5	1.3 ± 0.4	0.035
Day of Embryo transfer			
2nd Day	22 (12.5%)	9 (9.9%)	
3rd Day	90 (5.1%)	48 (52.7%)	0.356
4th Day	28 (15.9%)	9 (9.9%)	
5th Day	36 (20.5%)	25 (27.5%)	
Type of Trigger			
- GnRHa Trigger	85 (48.3%)	41 (45.1%)	0.799
- hCG Trigger	91 (51.7%)	50 (54.9%)	
Mode of ET			
- Frozen Transfer	127 (72.2%)	67 (73.6%)	0.615
- Fresh Transfer	49 (27.8%)	24 (45.1%)	
All Groups			
- GnRHa – Frozen ET	85(48.3%)	41 (45.1%)	
- hCG – Frozen ET	42 (23.9%)	26 (28.6%)	0.704
- hCG – Fresh ET	49 (27.8%)	24 (26.4%)	

GnRHa: GnRH agonist; hCG: human Chorionic Gonadotropin; SD: Standard deviation; *: Duration of infertility; FSH: Follicle Stimulating Hormone; LH: Luteinizing Hormone; E2: Es-tradiol Hormone; AFC: Antral Follicle Count; AMH: Anti-Mullerian Hormone; E2: Estradiol; MII: Metaphase 2; ET: Embryo Transfer; Values were presented as mean±SD or n, %, *: $p < 0.05$.

.
